# A Case of Scurvy in a Child: An Uncommon but Important Diagnosis to Consider

**DOI:** 10.7759/cureus.39369

**Published:** 2023-05-23

**Authors:** Aziza Elouali, Zohair El haddar, Yasser Bouabdella, Maria Rkain, Abdeladim Babakhouya

**Affiliations:** 1 Pediatrics, Faculty of Medicine and Pharmacy, Mohammed Ist University, Oujda, MAR; 2 Pediatrics, Mohammed VI University Hospital, Oujda, MAR

**Keywords:** child nutrition, vitamin c, ascorbic acid, case report, scurvy

## Abstract

Scurvy is a rare disease resulting from a prolonged ascorbic acid deficiency. It commonly affects individuals with low incomes and limited access to fresh fruits and vegetables. The diagnosis of scurvy can be challenging for clinicians due to the non-specific symptoms, resulting in extensive investigations and a delayed diagnosis. We report the case of a 14-year-old girl who presented with symptoms of pallor, bone pain, inability to walk, petechiae, ecchymosis of the lower limbs, irritability, and swollen, bleeding gums. Initially, the symptoms raised suspicions of hematopoietic malignancies, such as leukemia, and the patient underwent a series of invasive diagnostic exams before arriving at the correct diagnosis. Following a detailed dietary anamnesis with the parents, it became evident that the patient had a limited intake of fresh fruits and vegetables. A vitamin C blood test confirmed the diagnosis of scurvy. The prompt resolution of the clinical symptoms after the administration of vitamin C provided further confirmation of the diagnosis. This case highlights the importance of considering a patient's medical history and symptoms rigorously to avoid unnecessary, expensive, and invasive medical procedures, as well as to prevent potential misdiagnosis.

## Introduction

Scurvy is a rare disease that results from a prolonged deficiency of ascorbic acid, also known as vitamin C. Although rare, it is still reported in several case reports, particularly in individuals with low incomes and limited access to fresh fruits and vegetables [1]. The disease can be identified by a medical history indicating insufficient vitamin C intake for at least one to three months and a range of clinical manifestations, including cutaneous hemorrhagic syndrome, lower limb pain, and gingival bleeding. Scurvy can be easily misdiagnosed as it mimics other diseases such as hematopoietic malignancies and rheumatic diseases, leading to costly and excessive explorations [2,3]. This article describes a challenging case of scurvy in a 14-year-old girl who presented to a pediatric emergency with pallor, bone pain, hemorrhagic syndrome, and general asthenia, which initially mimicked a hematological disorder. She has progressed well under substitution treatment with vitamin C.

## Case presentation

We present the case of a 14-year-old girl admitted to our department with hemorrhagic syndrome, including gingival bleeding, purpuric lesions, and pallor, in addition to walking difficulties, myalgia, and bone pain in her lower limbs. The patient was the firstborn child of a non-consanguineous couple living in a rural village in Morocco with a low income. Her symptoms had been progressively worsening over the course of five months, during which she had experienced increasing myalgia and bilateral knee and ankle pain. She also experienced a general weakness due to anorexia and weight loss. The patient had no previous medical history of a similar eruption or trauma, and there was no known family history of hematological or systemic disease.

On physical examination, the patient was pale, cachectic, and irritable; there was no fever, hepatosplenomegaly, or lymphadenopathy. Vital signs were normal, with a blood pressure of 100/60 mmHg, a temperature of 37 °C, a peripheral pulse rate of 100 beats per minute, and a respiratory rate of 24 beats per minute. The patient weighed 20 kg (−3 standard deviation, SD) with a height of 132 cm (−3 SD). Due to the absence of previous measurements, the growth curve reconstruction was not possible.

**Figure 1:**
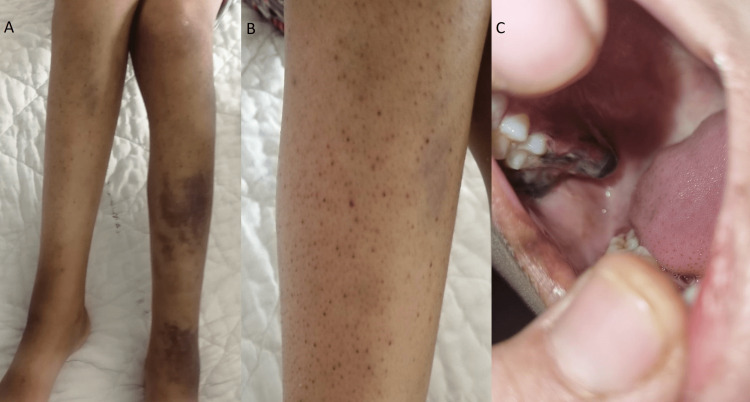
Clinical symptoms in our case (A) Purpuric rash and confluent ecchymoses on both legs, (B) purpuric skin lesions, (C) gingivitis with spontaneous bleeding

A musculoskeletal examination revealed a knee flexion contracture and lower limb edema, primarily on the ankles and right knee. The patient was unable to walk since she experienced pain upon joint mobilization. In addition, she had petechiae and confluent ecchymoses (Figure 1A-1B) on her legs, which had been developing for 20 days before admission. Examination of the oral cavity revealed gingival bleeding and bilateral inflammatory lesions along the upper gingival (Figure 1C).

The patient also exhibited behavioral symptoms, including lassitude and irritability. The patient's symptoms suggested acute leukemia or lymphoma. After conducting several blood tests, results showed microcytic hypochromic regenerative anemia (hemoglobin 5.7 g/dL), requiring a blood transfusion, a platelet count of 208,000/µL, and a white blood cell count of 5,070/µL. The bone marrow aspiration and blood smear did not indicate the presence of blasts. Renal function, liver function, muscle enzymes, and prothrombin time were all normal. However, the C-reactive protein was 13.3 mg/dL and the erythrocyte sedimentation rate (ESR) was 20 mm/h, which were both slightly elevated. A biopsy of the gingival lesions did not show any signs of malignancy. The computed tomography (CT) scan showed no abnormalities. After conducting these tests, we ruled out oncologic disorders. However, we suspected vasculitis, such as Henoch-Schönlein purpura, due to polyarthralgia, abdominal pain, and purpura. Another biopsy of the purpuric lesions did not show signs of vasculitis.

**Figure 2:**
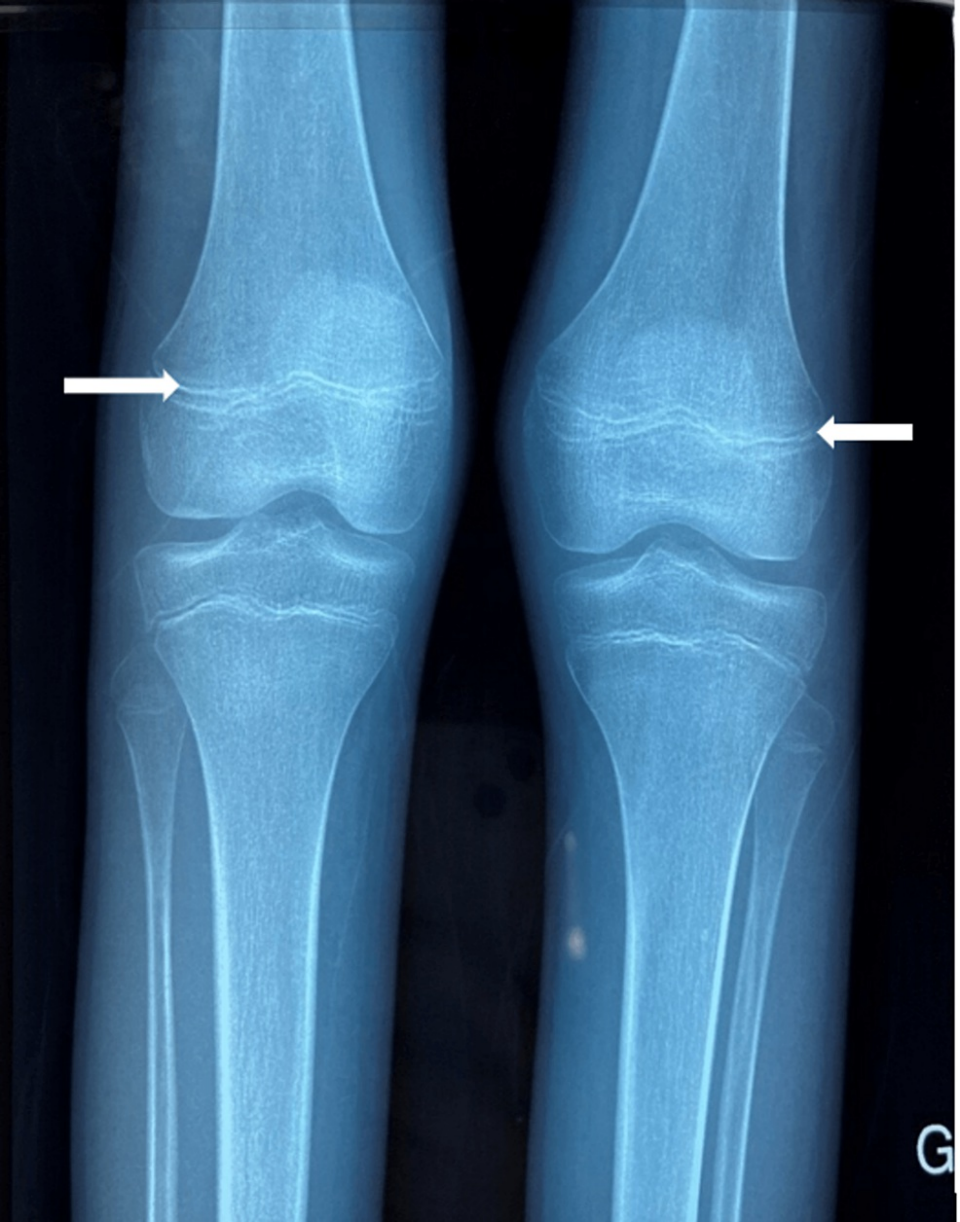
X-ray of the lower limbs showing symmetric dense metaphyseal bands of the femur

After a few days, scurvy caused by poor intake and/or absorption of vitamin C was suspected. We interviewed her parents to obtain a detailed dietary history, which revealed the girl's preference for tea and bread and a poor intake of fresh fruits and vegetables. The diagnosis was confirmed; the concentration of ascorbic acid showed an extremely low concentration of ascorbic acid, under 3 μmol/L. Additionally, radiographic imaging of the lower limbs revealed diffuse bone demineralization and thinning of the bone cortex of the fibula (Figure 2).

The results of other tests, including anti-transglutaminase antibodies, immunoglobulin A IgA, fecal occult blood, Helicobacter pylori, and fecal calprotectin, were all normal. The diagnosis of scurvy due to poor intake was confirmed, and treatment with vitamin C at a dose of 500 mg twice daily was started. We observed quick relief of the girl's pain in four days and a spectacular improvement in mucocutaneous lesions, gingival ulcers, and overall health after vitamin C administration. She gradually regained her walking ability.

## Discussion

Ascorbic acid, also known as vitamin C, is an essential, water-soluble vitamin that cannot be synthesized in the human body. It is involved in numerous physiological functions, such as the biosynthesis of carnitine and norepinephrine, amidation of peptide hormones, tyrosine metabolism, conversion of cholesterol into bile acids, and leukocyte function. In addition, vitamin C is a potent antioxidant that helps protect the body from oxidative stress, supports immune function, and facilitates iron absorption from the small intestine. Moreover, vitamin C is protective against several bacteria, including tetanus, diphtheria, and typhoid [4]. The primary dietary sources of vitamin C are fruits and vegetables. A deficiency of vitamin C leads to scurvy, a condition characterized by a defect in collagen synthesis that results in clinical signs observed in tissues containing collagen, including skin, cartilage, dentin, osteoid, and capillary blood vessels [5,6].

Scurvy has various manifestations, with the most common being musculoskeletal symptoms, which affect 80% of cases. These symptoms typically occur late and may include bone pain, arthralgia, myalgia, hemarthrosis, and muscular hematomas [7]. In infants, pseudo-paralysis and walking difficulties may also occur as a result of bleeding of soft tissues or joints, mimicking osteomyelitis or septic arthritis [7,8]. Our case presented with myalgia, swelling, and bone pain in the lower limbs, along with edema mostly at the ankles and an inability to walk. Dermatological symptoms may also be present, such as bruising, ecchymosis, petechiae, and perifollicular hemorrhages. Oral symptoms include swollen and inflamed gums, bleeding, and loosening of teeth [9,10]. The patient had a petechial rash, perifollicular purpura, and confluent ecchymosis on the legs. In addition, children with scurvy may experience systemic symptoms, including asthenia, lassitude, anorexia, failure to gain weight, and irritability [11].

A deficiency in vitamin C can lead to anemia due to bleeding (from the gastrointestinal or soft tissues), dietary deficiencies, intravascular hemolysis, and decreased iron absorption. In this case, the patient presented with hypochromic microcytic anemia with a hemoglobin level of 5.7 g/dL, requiring a red blood cell transfusion. Scurvy has specific radiological markers, including the "Frankel line" (dense metaphyseal bands), the "Wimberger ring sign" (a circular, opaque radiologic shadow surrounding epiphyseal centers of ossification), the "Pelkin spur" (metaphyseal spurs that result in a cupping of the metaphysis), and the "Trummerfeld zone" (lucent metaphyseal band underlying the Frankel line) [12]. While generalized osteopenia is not specific, it is a common finding. The clinical presentation of scurvy can vary widely and often mimic other systemic diseases, such as rheumatologic, neurologic, hematologic, and neoplastic conditions [12]. The patient in this case presented with weight loss, pallor, bone pain, petechiae, ecchymosis, upper jugal ulcerations, and hemorrhagic gingivitis. Based on these symptoms, she was immediately referred to the hematology-oncology unit and underwent costly and excessive investigations to rule out leukemia or lymphoma.

After an exhaustive evaluation, the diagnosis was ultimately revised to scurvy due to the child's poor dietary history and lack of fresh fruits and vegetables in her diet. Other nutritional deficiencies were also identified, including a deficiency in vitamin D and folic acid, requiring additional supplementation. To address the deficiencies, we initiated treatment with vitamin C at a dose of 500 mg twice daily and an appropriate nutritional diet. Individualization of the dose and duration of vitamin C treatment is crucial. The oral doses reported in the literature range from 100 mg to 300 mg to 4000 mg daily in divided doses. Treatment duration varies and should continue until full resolution of clinical symptoms, which can take anywhere from two weeks to seven months [13-14]. Upon treatment with vitamin C, spontaneous bleeding, oral symptoms, and constitutional symptoms typically improve within days, while skeletal changes and ecchymoses may take several weeks to resolve [15].

In this case, the diagnosis of vitamin C deficiency was based on clinical features, dietary history, and a low vitamin C level. Normal levels of AGA, anti-transglutaminase antibodies, IgA, IgG, and fecal calprotectin excluded gastrointestinal diseases as the cause of vitamin C deficiency. Following vitamin C supplementation, the child's pain improved rapidly, and there was a significant improvement in her mucocutaneous lesions, gingival ulcers, and overall physical condition. The child's walking ability gradually returned to normal.

## Conclusions

The presented case emphasizes the continued prevalence of scurvy in the pediatric population, especially among those who consume an inadequate amount of fresh fruits and vegetables. As pediatricians, we must remain vigilant regarding the prevalent symptoms of scurvy to ensure a prompt and accurate diagnosis of this potentially fatal disease. This can aid in preventing unnecessary invasive tests and ensuring appropriate care.
